# Digital technology and psychological happiness: the mediating roles of interpersonal relationships and employment situations

**DOI:** 10.3389/fpsyg.2024.1420511

**Published:** 2024-10-02

**Authors:** Wenxin Hu, Ziwen Zhang, Xinyue Qu, Yufei Mao

**Affiliations:** ^1^School of Economics and Management, Beijing Institute of Petrochemical Technology, Beijing, China; ^2^School of Labor Economics, Capital University of Economics and Business, Beijing, China

**Keywords:** psychological happiness, digital technology, influencing mechanism, interpersonal relationship, employment status

## Abstract

Psychological happiness represents the ultimate pursuit of human beings, and the impact of digital technology on psychological happiness is becoming increasingly significant in the era of the digital economy. Based on data from 2020 China Family Panel Studies (CFPS), this study constructs an empirical model that examines the effect and mechanism of digital technology on happiness. Additionally, this study investigates the heterogeneity and robustness of the impact of digital technology on happiness. The research conclusions are as follows: Firstly, digital technology can promote psychological happiness. When controlling for other factors, the marginal effect coefficient of digital technology is 0.031. Secondly, the effect of digital technology on personal happiness varies among different groups, particularly among women, young individuals, primary and college graduates, and rural residents. Furthermore, as absolute income increases, the happiness effect of digital technology diminishes. Thirdly, in terms of the influencing mechanism, digital technology indirectly affects individual happiness by influencing health status, interpersonal relationships, employment situations and income levels. Specifically, digital technology negatively impacts personal health, interpersonal relationships, and agricultural work, while positively impacting family relationships, non-agricultural employment, absolute income and relative income. Digital technology affects happiness through these channels indirectly. Based on the study results, it is proposed that efforts should be made to enhance the development of digital technology infrastructure in remote rural areas, reduce the financial burden associated with digital technology, and promote the digital technology ecosystem. Moreover, providing online services, such as e-commerce, travel reservations, and digital financial management, can improve access to digital technology in rural areas and contribute to increased happiness levels. Simultaneously, there is a need to strengthen digital skills training, particularly among vulnerable populations such as the elderly and rural residents, to improve their proficiency in digital technologies. This can be achieved through the integration of additional educational resources, thereby facilitating cost-free digital technology training and guidance. Meanwhile, it is essential to vigorously develop the new economy and innovative employment models, create job opportunities, foster entrepreneurial prospects, and improve income levels to enhance individual well-being.

## Introduction

Aristotle once said: “Happiness is the best,” that is, the ultimate goal of life is to seek happiness. With the development of social economy in the new era, residents’ pursuit has shifted from predominantly material and cultural desires to an ardent pursuit for life quality, and “improving people’s happiness” is becoming the main theme of people’s livelihood issues (Munene et al., 2022; Saida et al., 2021). In recent years, the word “happiness” frequently appears in various news, government reports and academic research. The enhancement of happiness is not only a primary objective of economic development and governmental initiatives, but also yields numerous tangible advantages for individuals (Srivastava et al., 2022; Yuan et al., 2021). The report of the 19th National Congress of the Communist Party of China in 2017 clearly pointed out that “people’s happiness should be more substantial, more secure and more sustainable.” In 2021, Premier Li Keqiang further pointed out that government should effectively implement livelihood projects and continuously enhance the “happiness index” of people’s livelihood. Subsequently, the term “happiness” gained prominence in the discourse of the Tenth Five-Year Plan and government reports and well-being of residents emerged as a key focal point in the government’s policy agenda. According to the United Nations Global Happiness Index Report 2020, the happiness index of China residents is 5.585, ranking 72nd in the world, which is lower than the global average happiness level. The World Happiness Report suggests that despite China’s rapid economic growth since the 1990s, there has been no significant increase in the country’s happiness index. Instead, there has been a fluctuation, with an initial decrease followed by an increase. This trend can be attributed to the changes in the employment and social security system during the economic transformation (Easterlin et al., 2017). How to enhance residents’ well-being and address their needs more effectively has become a crucial guarantee for fostering the harmonious and sustainable growth of the social economy.

In the era of digital economy, digital technology is assuming a growing significance in individuals’ daily lives, offering enhanced convenience and efficiency to support and assist them (Ai-Rawashdeh, 2021). Since the 21st century, digital technology and information technology have flourished around the world, and have penetrated into all areas of people’s daily lives, subtly changing people’s ways of study, work, social interaction and leisure (Karabacak and Gen, 2019). According to National Population Census of China Database, the proportion of urban population in China is 45.4%, and that of rural population is 54.6%. In rural areas of China, due to the lack of digital infrastructure and low education levels, the rural utilization rate of digital technology is relatively low. Meanwhile, the rural population in China accounts for 54.6% of the total population, resulting in national utilization rate of digital technology is not high. The network economy has emerged as a key driver of national development and digital technology applications such as instant messaging, social platforms, mobile payment and online entertainment are reshaping the lifestyle of residents. Based on the theories of technological progress and labor economics, the advancement of digital technology is expected to enhance the convenience of daily life and optimize work efficiency. Furthermore, it is projected to elevate social interaction and income levels, ultimately contributing to the overall increase in happiness and well-being (Krueger, 1993; Kuhn and Mansour, 2014). Considering the advancing digital economy and the expanding depth and breadth of digital technology application, can digital technology improve personal happiness? What is the influence mechanism? In which groups is the happiness effect of digital technology more obvious? How to prevent internet addiction and maximize the benefits of online resources? These studies hold significant practical implications for improving personal happiness under the background of digital economy.

The study of happiness belongs to the interdisciplinary research field, which first started in psychology and sociology, and then gradually attracted the attention of economics (Hood et al., 2021; Rossouw et al., 2021). Happiness economics originated from the well-known “Easterlin Paradox,” which primarily examines the correlation between national income and happiness. It posits that happiness is influenced not only by increases in absolute income but also by comparisons of relative income and external factors beyond income. Based on the research from the perspective of economic income, this paper primarily explores the variations in happiness on absolute income, relative income, social status, values and consumption. Based on the research of non-income factors, it focuses on the demographic factors, such as gender, age, household registration, health, marriage and education, as well as variables such as work status, family factors and interpersonal factors on happiness (Mohammadi et al., 2022; Bucciol and Burro, 2022; Mackerron, 2012). The latest research also delves into the relationship between economic globalization, public services and other policy factors (Saida et al., 2021). Nevertheless, with the rapid advancement of digital technology, it is worth noting that scholarly attention towards the impact of digital technology on happiness is increasingly gaining favor (Graham and Nikolova, 2013), but the research conclusion is controversial, and there is a lack of analysis on the causal mechanism of digital technology’s impact on happiness, as well as the heterogeneity of individual happiness effects of digital technology.

Different from the existing literature, the contributions of this paper are as follows: Firstly, previous studies neglected the study of different mechanisms, and most of them only focused on interpersonal relationship. This paper discusses the indirect influence of digital technology on happiness from the perspectives of health status, family and interpersonal relationship, employment status and income levels. Secondly, previous studies have missed the heterogeneous influence of digital technology on their happiness in different situations. This paper investigates the happiness effect of digital technology among people varying ages, genders and academic qualifications. Thirdly, prior studies have overlooked the endogenous issue related to digital technology, consequently impacting the reliability of their research findings. In this paper, the CMP model is used to discuss the endogenous problems, and the robustness of the research conclusions is tested by 2018 CFPS data.

## Literature review and theoretical framework

### Direct impact of digital technology on psychological happiness

With the advancement of the digital economy and the enhancement of material living standards, the influence of digital technology on residents’ lives has garnered significant attention (Zhu and Leng, 2018; Zuo, 2021). Scholars have conducted extensive discussions on digital technology and its impact on interpersonal communication and employment methods. Happiness, as one of the important indicators of subjective well-being in people’s lives (Tella et al., 2003), understanding the mechanism of digital technology’s impact on happiness is of great practical significance for building a harmonious society and promoting people’s welfare. The study of digital technology and happiness first began from the perspectives of psychology and sociology (Lohmann, 2013). Research in psychology examines the correlation between digital technology and mental health, loneliness, and life satisfaction (Ulukan and Ulukan, 2021; Kraut and Burke, 2015). On the other hand, research in sociology delves into the connection between digital technology and factors like interpersonal communication, social networks, and social support, which subsequently influence personal happiness (Lee et al., 2011). Later on, economic research utilizing empirical models to examine the relationship between digital technology and happiness has been extensively discussed. It has been confirmed that digital technology enhances personal happiness. For example, using data from the 2008 European Values Survey, Pénard et al. (2013) found that digital technology had a positive impact on personal happiness, especially for young people or those with low incomes. Zhu and Leng (2018) demonstrated that the utilization of digital technology substantially enhanced residents’ subjective well-being. This impact remained significant even after incorporating other control variables and considering regional disparities. Moreover, the positive effect on happiness among rural residents surpasses that of urban residents. The reason why digital technology can bring about happiness is twofold. Firstly, digital technology offers online entertainment like literature, music, videos, games, and various applications and services, which directly or indirectly benefit individuals. Secondly, digital technology provides new communication channels such as instant messaging, microblogging, social networking sites, which enhance interpersonal emotional communication, facilitate contact and alleviate life pressures. In addition, people obtain information by browsing online news and utilizing search engines. Online business activities such as online shopping, online payment, and travel booking bring convenience to life, which positively affects individual happiness.

Other studies pointed out that digital technology was a double-edged sword. Kraut and Burke (2015) noted that while connectivity can broaden access to information and enhance personal happiness, it can also have adverse effects. Excessive use of digital technology can lead to Internet addiction, social isolation and psychological stress. Graham and Nikolova (2013) used Gallup World Poll data to explore the impact of information technology access on well-being. They found that overall use of phones, television and digital technology had a positive impact on happiness, but this impact had diminishing marginal returns and also brought some negative psychological anxiety. Nie et al. (2016) found that digital technology has a negative effect on happiness. Weiser (2001) argued that utilizing the emotion regulation function of digital technology decreases happiness by reducing social integration. Utilizing the information gathering function of digital technology increases social integration and thus has a positive effect on happiness. A study showed that the duration of digital technology was negatively correlated with happiness. Information collection through digital technology has a positive effect on happiness, while using digital technology for emotional expression does not have a significant effect on happiness. Shen et al. (2014) concluded that access to information through digital technology and online dating had a significant positive effect on happiness. Conversely, digital technology entertainment had a significant negative effect on happiness. Additionally, social support was found to play a mediating role between digital technology and happiness.

### The indirect impact of digital technology on psychological happiness

The influence of digital technology on happiness is complicated. In addition to directly affecting happiness, numerous intermediary or moderating variables May play a role in indirectly affecting happiness. In the study of happiness economics, the factors that affect happiness are divided into economic factors and non-economic factors, among which, health status, family and interpersonal relationships, work factors and income factors are deemed crucial in determining personal happiness (Mackerron, 2012), and digital technology May indirectly affect happiness by influencing these intermediary variables.

Health status has a strong positive impact on happiness (Mohammadi et al., 2022), and digital technology will affect personal psychological and physical health, subsequently impacting their overall happiness. With regard to the relationship between digital technology and health status, early studies mainly focused on the problem of Internet addiction caused by excessive usage of digital technology. It is hypothesized that the utilization of digital technology May diminish individuals’ mental health level, amplify feelings of social isolation, and potentially trigger psychological ailments such as depression (Kraut and Burke, 2015). At the same time, unhealthy digital technology habits and prolonged screen time are also likely to adversely affect physical well-being, for example, resulting in physical discomfort such as reduced visual acuity and neck pain (Zheng et al., 2016). However, some studies believe that using digital technology has a better mental health level and a higher sense of happiness than not using it. The interpersonal communication and mass communication functions of digital technology can interfere with personal health, and using digital technology can contribute to the promotion of health knowledge and improve health literacy, thus changing their own health attitudes and behaviors.

Interpersonal communication plays a pivotal role in fostering individuals’ happiness (Doley and Anbarasan, 2021). Prior research has demonstrated that engaging in communication with friends and family or participating in group activities, can enhance the quality of social support and interpersonal connections, ultimately leading to heightened happiness (Diener et al., 2013). In reality, digital technology not only facilitates the expansion of weak ties by enabling the establishment of new connections through social media, but also aids in sustaining existing close relationships, thus enabling individuals to receive heightened emotional support (Ellison et al., 2014). Simultaneously, the openness and inclusivity of digital technology broaden the realm of interpersonal communication, enhancing its extensiveness and autonomy. Moreover, by concealing identity and lacking physical presence, it diminishes interpersonal trust and emotional connection in real life (Shen et al., 2014). Nevertheless, as individuals increasingly rely on digital technology and scholarly investigation deepens, researchers have observed that digital technology diminishes individuals’ temporal time and availability in face-to-face interactions. The interpersonal network formed through digital means is often deemed unreliable, thus potentially impeding genuine communication. This phenomenon can be attributed to a substitution dynamic between digital technology and authentic interpersonal communication. Specifically, digital technology consumption displaces the time allocated for face-to-face social engagements, while low-quality network connections supplant high-quality interpersonal relationships. These trends collectively exert a notable adverse influence on individuals’ interpersonal connections. Additional studies have confirmed that despite visual communication can be achieved through the Internet, face-to-face interactions continue to hold significance in developing long-term relationships and mutual support (Lee et al., 2011).

Employment status is a crucial variable in determining happiness (Wolfe and Patel, 2021; Bucciol and Burro, 2022), and digital technology will significantly affect personal employment decisions, thereby impacting happiness. Feldman and Klaas (2002) found that managers and professionals are more inclined to utilize digital technology for job searches, and the efficiency of this search method exceeds that of traditional newspaper and telephone advertisements. Pierewan and Tampubolon (2014) used European Social Survey and found that employing digital technology to cope with unemployment amidst the financial crisis substantially enhanced individual happiness. Scholars have also conducted empirical studies to investigate the effects of digital technology on employment, highlighting that digital technology can notably enhance the job security and elevate the job quality of floating population. Further research has indicated that accessing professional and business information via digital technology can increase the likelihood of entrepreneurial ventures (Karabacak and Gen, 2019), particularly in the context of opportunistic entrepreneurship. This is because, on the one hand, using digital technology for online job applications helps to reduce market information asymmetry, enhances the efficiency of job seekers in submitting resumes and browsing recruitment websites (Kuhn and Mansour, 2014). On the other hand, digital technology skills also mean higher investment and accumulation of human capital, and the resulting signal function aids employers identify potential individuals with higher labor productivity. In addition, the digital technology platform not only alleviates physical job demands but also enhances employment flexibility by reducing commuting time and mitigating workplace disruptions (Bloom et al., 2015).

Income is recognized as a pivotal determinant of subjective well-being, with an extensive body of empirical literature exploring the intricate interplay between relative income, absolute income, and happiness (La et al., 2021), and it is posited that digital technology will have an impact on the absolute income and relative income. Judging from the relationship between digital technology and absolute income, existing studies have demonstrated a positive correlation between digital technology and individual wage income (Li and Xie, 2017). On the one hand, leveraging digital technology in the workplace has the potential to enhance operational efficiency and stimulate individuals to participate in more innovative and creative professional endeavors. On the other hand, the swift advancement of digital technology has led to a heightened requirement for specialized workforce, resulting in an amplified “complementary effect” between technological evolution and skilled labor. This synergy has subsequently led to elevated wage premiums for individuals proficient in these digital skills (Levy and Murnane, 1996). Several research studies have indicated that the wage premium associated with digital technology extends beyond its utilization in the workplace, as engaging with digital technology in domestic settings can also enhance individuals’ earnings by fostering the development of information technology human capital (Krueger, 1993; Dimaggio and Bonikowski, 2008). In the context of the correlation between digital technology and relative income, initial investigations have posited that television viewing represents an additional factor in elucidating the “happiness-income” paradox. This is attributed to the notion that television consumption May result in heightened exposure to information, leading to an increase in materialistic aspirations and anxiety levels, consequently diminishing the impact of income on personal happiness (Bruni and Luca, 2006). It is identified that digital technology broadens the sphere of income comparisons, subsequently influencing individuals’ self-perception and evaluations of their socio-economic status, thus indirectly impacting their subjective happiness.

**Figure 1:**
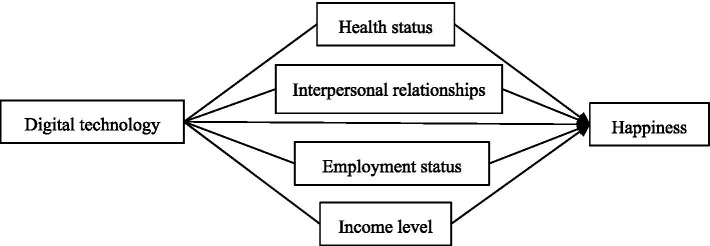
The theoretical framework.

According to the literature review, based on the theory of happiness economics, the main factors affecting happiness are health status, family and interpersonal relationships, employment status, absolute income and relative income. The influence mechanism of digital technology on happiness is complicated. On the one hand, digital technology can directly contribute to individual happiness, including entertainment, information access, and life convenience, can directly enhance personal happiness. On the other hand, digital technology can indirectly affect happiness by changing personal health status, interpersonal relationship, employment status and income level. Therefore, when analyzing the influence of digital technology on happiness, it is necessary to not only consider the overall effect on happiness, but also to delve into the intermediary variables, in order to gain a more profound understanding of the mechanisms by which digital technology influences happiness. The theoretical framework constructed in this paper is shown in Figure 1.

## Materials and methods

### Data sources and variable descriptions

The empirical analysis utilizes data from the 2020 China Family Panel Studies (CFPS). The China Family Panel Studies (CFPS) is a large-scale comprehensive social science research project led by the Institute of Sociology, Chinese Academy of Social Sciences. The project aims to comprehensively understand China’s social changes and their impact on family structure, population health, education level, economic status, and other aspects through long-term tracking surveys. The survey scope of CFPS includes a comprehensive sample of 32,669 adults, which covers 25 provinces and regions in China, striving to reflect the socio-economic characteristics and cultural diversity of different regions in China. The survey questions cover a wide range of areas, including but not limited to the basic information of family members, marriage and childbirth, education and occupation, health and medical care, social relations and networks, family income and property, living conditions and community environment, etc.

The cultural background of CFPS is that China is undergoing rapid socio-economic transformation, which not only changes the frequency of residents’ use of digital technology, but also has an impact on residents’ happiness and other aspects. Specifically, this study focuses on the working-age population (16–65 years old) as the sample. The missing samples of key variables such as digital technology and happiness were cleared, leading to a final sample size of 10,161 participants. The paper selects and provides explanations for the key research variables as follows:

1. Explained variable: psychological happiness. This article selects the indicator “How happy do you feel?.” The measurement of happiness is based on a 5-point scale, where 1 represents very unhappy and 5 represents very happy. The higher the score, the higher the level of happiness.
2. Explanatory variable: digital technology. This article selects the indicator “Do you use the Internet in your daily life and work?” from the questionnaire as a variable for the digital technology status, where using digital technology is assigned a value of 1, otherwise it is 0.
3. Mediating variables: health status, interpersonal relationships, employment status, income level. Meanwhile, control variables such as personal characteristics and regional characteristics were also included in the study. “Gender,” “age,” “registered permanent residence,” “nation,” “marital status,” and “education level” were selected as variables to measure personal characteristics. The variables “urban/rural classification” and “province or municipality directly under the central government” were selected to measure regional characteristics. The specific variables selected and their meanings are shown in Table 1.

**Table 1:** Variables and descriptions.

Dimensions	Variables	Variable interpretation
Happiness	Psychological happiness	Very unhappy = 1, unhappy = 2, average = 3, happy = 4, very happy = 5
Digital technology	Whether or not you use the internet	Yes = 1, no = 0
Intermediary variable	Health status	Unhealthy = 1, Fair = 2, Fairly healthy = 3, Very healthy = 4, Very healthy = 5
	Family relations	Frequency of interaction and contact with relatives not living with the household (no interaction = 1, infrequent interaction = 3, occasional interaction = 3, frequent interaction = 4)
	Interpersonal relationship	Number of self-rating scores for personal relationships (minimum = 0, maximum = 10)
	Employment status	Non-farm jobs = 1, farming jobs = 2, unemployment = 3
	Absolute income	Logarithmic net *per capita* household income last year
	Relative income	Relative position of personal income in the locality (very low = 1, very high = 5)
Personal characteristic	Gender	Male = 1, female = 0
	Age	Actual age (in years)
	Registered permanent residence	Urban household registration = 1, Rural household registration = 0
	Nation	Han nationality = 1, non-Han nationality = 0
	Marital status	Married = 1, unmarried = 0
	Education level	Uneducated = 1, Primary education = 2, Junior high school education = 3, High school/technical secondary school = 4, College degree = 5, Bachelor degree = 6, Master degree or above = 7
Regional characteristics	Urban and rural classification	Urban = 1, rural = 0
	Province	Dummy variable for the province or municipality where the survey was conducted

### Econometric model

#### The impact of digital technology on psychological happiness

To examine the impact of digital technology on happiness, this paper utilizes an ordered Probit model for econometric analysis. The benchmark model is set as follows:


(1) $$\mathrm{Happiness}=\alpha +\mathrm{\beta Digital}\;\mathrm{Technology}+\gamma X_{1}+\lambda X_{2}+\varepsilon$$

In Equation 1, the variable *Happiness* represents the level of happiness, while the variable *Digital Technology* indicates whether or not digital technology is used. The control variables refer to the existing literature. *X*_1_ represents personal characteristics such as gender, age, registered permanent residence, nation, marital status, and education level. *X*_2_ represents regional characteristics such as urban and rural classification and province of residence. In this study, *β* represents the marginal effect of digital technology on happiness, while *γ* and *λ* represent the coefficients of influence for personal and regional characteristics. The term *ε* represents the random perturbation.

#### The impact of different digital technology applications on psychological happiness

According to the CNNIC report, individuals use digital technology for various purposes, mainly including online learning, online work, online socializing, online entertainment, and online business.

Relevant studies show that there are differences in the application of digital technology among different gender individuals, with men using digital technology more often for online office and online games, etc., while women are more inclined to use digital technology for social chatting, online shopping and learning and communication. In order to analyze the effect of different digital technology applications on happiness, this paper adds the interaction term of digital technology and the frequency use of different digital technology applications on the basis of the baseline model, and also distinguishes between the full sample and the different gender samples, and constructs the model as shown in Equation 2:


(2) $$\mathrm{Happiness}=\alpha +\beta_{0}DT+\beta_{i}DT\times IU_{i}+\gamma X_{1}+\lambda X_{2}+\varepsilon$$

In Equation 2, *IU*_i_ denotes the usage frequency of different digital technology applications, such as digital technology for study, work, socialization, entertainment and business activities, etc., and *β*_i_ denotes the marginal effect of different digital technology applications on happiness.

#### The test of the influence mechanism of digital technology on psychological happiness

In order to further explore the influence mechanism of digital technology on happiness, this paper adding mediating variables in the benchmark model and test the influence mechanism of digital technology on happiness, and constructs the model as shown in Equation 3:


(3) $$\mathrm{Happiness}=\alpha +\beta_{0}DT+\beta_{i}\mathrm{mediato}r_{i}+\gamma X_{1}+\lambda X_{2}+\varepsilon$$

Equation 3 defines *mediator*_i_ as mediating variables, such as self-assessed health status, family and interpersonal relationships, employment status, and income level. The symbol *β*_i_ represents the marginal effect of each mediating variable on happiness.

### Descriptive statistics

Table 2 presents the descriptive statistics of the key variables. The research focuses on the working-age population aged 16–65. After excluding samples with missing key variables such as digital technology and happiness, 10,355 valid samples were obtained, with 6,544 (63.2%) of them have used digital technology applications. In rural areas of China, the low usage rate of digital technology among residents is mainly influenced by various factors. Firstly, inadequate infrastructure is one of the main obstacles restricting the popularization of digital technology in rural areas. Compared with urban areas, the broadband network coverage in rural areas is limited, and the Internet access speed is slow, leading to a greatly reduced experience of using digital devices. Secondly, economic conditions limit the ability of rural residents to purchase and maintain digital devices. The lower income level makes it difficult for many families to afford the cost of devices such as smartphones and computers, thereby limiting the scope of digital technology applications. Furthermore, differences in educational levels are also a major factor. Due to uneven distribution of educational resources, residents in rural areas generally lack digital skills training and information technology education, which directly affects their ability and willingness to use digital technology. In addition, cultural and social habits have also hindered the popularization of digital technology to some extent. Some rural residents may be more inclined to rely on traditional ways of communication and information acquisition, with relatively lower acceptance of new technologies.

**Table 2:** Descriptive statistics of variables.

Description of variables	Full sample	Digital technology	Non-digital technology	*T*-test
Happiness	3.845 (0.990)	4.125 (0.900)	3.690 (1.025)	0.435***
Health status	3.105 (0.008)	3.425 (0.012)	2.958 (0.010)	0.467***
Family relations	3.438 (0.005)	3.531 (0.008)	3.395 (0.007)	0.136***
Interpersonal relationship	7.242 (0.012)	7.203 (0.019)	7.260 (0.015)	−0.057**
Non-agricultural work	0.495 (0.003)	0.799 (0.005)	0.355 (0.004)	0.444***
Farming	0.365 (0.003)	0.099 (0.003)	0.488 (0.004)	−0.389***
Unemployed	0.139 (0.002)	0.102 (0.004)	0.156 (0.003)	−0.054***
Absolute income	9.144 (0.007)	9.491 (0.011)	8.982 (0.008)	0.509***
Relative income	2.529 (0.006)	2.549 (0.01)	2.520 (0.008)	0.029**
Gender	0.528 (0.500)	0.510 (0.478)	0.485 (0.502)	0.025***
Age	42.48 (12.37)	35.24 (10.02)	47.02 (11.28)	−11.78***
Registered permanent residence	0.402 (0.448)	0.641 (0.483)	0.312 (0.413)	0.329***
Nation	0.935 (0.263)	0.953 (0.235)	0.902 (0.285)	0.051***
Marital status	0.868 (0.347)	0.773 (0.426)	0.905 (0.292)	−0.132***
Uneducated	0.201 (0.417)	0.031 (0.136)	0.296 (0.466)	−0.265***
Primary education	0.235 (0.431)	0.141 (0.329)	0.281 (0.455)	−0.140***
Junior high school education	0.316 (0.463)	0.352 (0.472)	0.283 (0.458)	0.069***
High school/technical secondary school	0.151 (0.362)	0.263 (0.437)	0.103 (0.329)	0.160***
College degree	0.063 (0.248)	0.149 (0.352)	0.012 (0.118)	0.137***
Bachelor degree	0.047 (0.193)	0.102 (0.313)	0.002 (0.061)	0.100***
Master degree or above	0.004 (0.062)	0.008 (0.083)	0.0002 (0.014)	0.006***
Urban and rural classification	0.478 (0.501)	0.637 (0.478)	0.395 (0.603)	0.242***
Sample size	10,355	6,544	3,811	–

Data in the table are sample means and data in parentheses are standard deviations. ***, **, and * Indicate significant at the 1, 5, and 10% levels, respectively.

The descriptive statistics reveal that individuals who use digital technology have an average happiness score of 4.124, while those who do not have an average score of 3.690. The data indicates that individuals who use digital technology report significantly higher levels of happiness than those who do not. Regarding health and interpersonal interactions, individuals who use digital technology tend to have better health and more frequent contact and communication with their families. However, their scores for interpersonal interactions are relatively lower. Individuals who utilize digital technology have a higher proportion of non-farming jobs and a lower percentage of farming or unemployment. Their absolute and relative income levels are also significantly higher than those who do not use digital technology. Furthermore, a higher proportion of digital technology users are young, single, and urbanized.

## Empirical analysis

### The overall impact of digital technology on psychological happiness

Firstly, this section examines the influence of digital technology on happiness. Table 3 presents the estimation results of the benchmark model, and the Wald test of the equation is significant at the level of 1%, which confirms the rationality of the measurement model. After adding digital technology variables and provincial virtual variables to the equation in the first column, the marginal effect coefficient of digital technology is 0.071 and is significant at the level of 1%. The equations in column (2), column (3) and column (4) sequentially add individual characteristic variables such as gender, age, household registration, nationality, marital status and education level, regional characteristic variables such as urban–rural classification. The regression result of column 4 shows that the marginal effect coefficient of digital technology is 0.031, which is significant at the level of 1%, indicating that when other factors are controlled. This shows that digital technology exerts a notable and positive influence on happiness.

**Table 3:** Benchmark model of the influence of digital technology on happiness.

Variable	(1)	(2)	(3)	(4)
Digital technology	0.071*** (0.004)	0.048*** (0.006)	0.039*** (0.005)	0.031*** (0.006)
Gender		−0.036*** (0.004)	−0.030*** (0.003)	−0.030*** (0.003)
Age		−0.019*** (0.003)	−0.018*** (0.004)	−0.019*** (0.003)
Age squared/100		0.024*** (0.001)	0.026*** (0.003)	0.023*** (0.003)
Registered permanent residence		0.045*** (0.005)	0.036*** (0.006)	0.029*** (0.005)
Nation		−0.016 (0.011)	−0.018 (0.013)	−0.018 (0.011)
Marital status		0.157*** (0.009)	0.160*** (0.007)	0.161*** (0.008)
Primary education			0.027*** (0.010)	0.024*** (0.009)
Junior high school education			0.037*** (0.007)	0.035*** (0.009)
Senior high school/Technical secondary school			0.040*** (0.010)	0.042*** (0.011)
College degree			0.078*** (0.013)	0.079*** (0.012)
Bachelor degree			0.067*** (0.013)	0.063*** (0.012)
Master degree or above			0.054 (0.037)	0.053 (0.036)
Urban and rural classification				0.012* (0.005)
Virtual variables of provinces	Control	Control	Control	Control
Wald χ^2^	1788.09***	2643.80***	2359.04***	2336.20***
Observed value	10,355	10,355	10,355	10,355

The standard error in brackets in the table is robust, and ***, **, and * indicate significant at 1, 5, and 10% levels, respectively. The reported value is the average marginal effect of each variable on happiness (the happiest = 5) and the corresponding robust standard error.

From the estimation results of individual characteristic variables, it can be seen that age and happiness show a U-shaped trend, indicating that as age increases, the probability of the happiest person decreases first and then increases. Gender exhibits a statistically significant negative effect on happiness, revealing that women tend to report higher levels of happiness compared to men. The negative coefficient associated with household registration implies that individuals with urban household registration are more likely to experience happiness than their rural counterparts. The coefficient of marital status is significantly positive, indicating that the happiness of married groups is higher than that of unmarried groups. Using those who have not attended school as a reference group, the probability of happiness is significantly higher for individuals with primary, junior high, high school/technical secondary, college, and undergraduate education. However, a master’s degree has no significant impact on individual happiness. Interestingly, the attainment of a master’s degree does not yield a significant impact on individual happiness levels. The coefficient of urban–rural classification is significantly positive, indicating that the probability of happiness of urban residents is significantly higher than that of rural residents.

### Heterogeneity of the impact of digital technology on individual happiness

In the previous paper, we analyzed the impact of digital technology on happiness and determined that it can improve individual happiness. However, this analysis only represents the average impact of digital technology on happiness and does not take into account individual differences. For this purpose, this paper examines the heterogeneity of the impact of digital technology on happiness by incorporating interaction terms of digital technology with gender, age, education level.

The results in Table 5 indicate that the coefficient of the interaction term between digital technology and gender in column 1 is significantly negative. This suggests that the use of digital technology has a greater impact on women’s happiness than on men’s, implying that women can gain more happiness by using digital technology. Possible reasons for this are that women’s utilization of digital technology for learning, work, and entertainment activities can enhance happiness. Studies have also confirmed that the application and popularization of digital technology have a significant effect on promoting women’s employment and income, indirectly increasing their sense of well-being. The interaction term coefficient between digital technology and age in Equation 2 is significantly negative. This suggests that digital technology has a greater impact on improving the happiness of young people. This may be due to the fact that young people are exposed to digital technology at an early age, possess advanced digital technology skills, and can enhance and enjoy their lives through its utilization. The coefficient of the interaction term in Equation 3 is significantly positive for digital technology with primary school education and tertiary education and above, but the coefficient of digital technology with secondary school education is not significant, indicating that the use of digital technology significantly enhances the well-being of those with primary school and college degree and above. The interaction term coefficient between digital technology and rural–urban categorization in column 4 is significantly negative. This suggests that the use of digital technology has a greater impact on the happiness of rural residents than on urban residents. The delayed development of digital technology in rural areas results in a delayed start for accessing information, online socializing and e-commerce. Once implemented, these technologies can bring greater satisfaction and fulfillment to rural residents. In column 5, the coefficient of the interaction term between digital technology and absolute income is significantly negative. This suggests that as income increases, the happiness promotion effect of digital technology is decreasing, and digital technology diminishes the positive effect of absolute income on happiness, which is consistent with previous research (Table 5).

**Table 4:** Influence of digital technology on intermediate variables.

Variable	(1)	(2)	(3)	(4)		(5)	(6)
	Health condition	Family relations	Interpersonal relationship	Non-farming employment	Farming employment	Absolute income	Relative income
Digital technology	−0.007* (0.004)	0.053*** (0.008)	−0.069** (0.032)	0.142*** (0.007)	−0.118*** (0.007)	0.246*** (0.017)	0.003** (0.001)
Control variable	Control	Control	Control	Control	Control	Control	Control
*F* value/Wald χ2	4499.26***	3780.76***	11.05***	14281.16***		200.99***	3802.43***
Observed value	23,465	23,358	23,446	23,465		21,397	22,662

Models (1), (2), and (6) use oprobit model, and the table reports the marginal effects of digital technology on very healthy = 5, frequent interaction = 4, and high income = 5, respectively. Models (3) and (5) use OLS model. Model (4) uses mlogit model, and the marginal effects of digital technology on non-farming employment and arming employment are reported in the table. Control variables in our study conclude personal characteristics and regional characteristics. Among them, “Gender,” “age,” “registered permanent residence,” “nation,” “marital status,” and “education level” were selected as variables to measure personal characteristics. The variables “urban/rural classification” and “province or municipality directly under the central government” were selected to measure regional characteristics. Due to space limitations in the paper, this table list the results for the core variables, do not list the results for the control variables.

**Table 5:** Heterogeneity analysis of the impact of digital technology on happiness.

Variable	(1)	(2)	(3)	(4)	(5)
Digital technology	0.025*** (0.009)	0.067*** (0.023)	−0.042 (0.034)	0.026*** (0.01)	0.182*** (0.053)
Digital technology* gender	−0.021** (0.010)				
Digital technology* age		−0.001** (0.001)			
Digital technology* Primary education			0.065* (0.037)		
Digital technology* Secondary school education			0.050 (0.035)		
Digital technology* College and above			0.099** (0.04)		
Digital technology* Urban and rural classification				−0.022* (0.011)	
Digital technology* Absolute income					−0.018*** (0.006)
Control variable	Control	Control	Control	Control	Control
Wald χ^2^	4635.09***	4635.25***	4649.84***	4649.84***	4637.96***
Observed value	20,666	20,666	20,666	20,666	20,666

Mediating and control variables are added to the analysis. ***, **, and * indicate significant at the 1, 5, and 10% levels, respectively. Control variables in our study conclude personal characteristics and regional characteristics. Among them, “Gender,” “age,” “registered permanent residence,” “nation,” “marital status,” and “education level” were selected as variables to measure personal characteristics. The variables “urban/rural classification” and “province or municipality directly under the central government” were selected to measure regional characteristics. Due to space limitations in the paper, this table list the results for the core variables, do not list the results for the control variables.

### Robustness tests for 2018 CFPS data

To ensure the robustness of the conclusions drawn from the analysis above, this paper utilizes the 2018 CFPS data to retest the impact mechanism. Equation 1 shows the baseline model, which includes personal and regional characteristics as control variables, yields a coefficient of 0.032 for the impact of digital technology on happiness, which is significant at the 1% level. The coefficient of Equation 2 is positive after adding the health status variable, and the coefficient of digital technology increases. This suggests a negative relationship between digital technology and health status, which is consistent with previous findings. The coefficients in Equation 3 are positive after adding the family relationship variable. Additionally, the coefficients of digital technology have decreased, which confirms that digital technology can enhance the frequency of communication with family members. The coefficient of digital technology in column 4 decreases after adding the interpersonal variable, indicating that digital technology favors the improvement of interpersonal relationships, which is contrary to the previous conclusion. The penetration rate of mobile digital technology was still low, and people’s use of digital technology devices such as computers and cell phones for communication and contact contributed to the establishment of interpersonal networks, and has not yet had a negative impact on interpersonal interactions in reality. Equations 5–7 add variables for employment status, absolute income and relative income. The coefficients for digital technology have decreased, consistent with previous findings. This suggests that digital technology has a significant impact on personal employment decisions and income levels. After adding absolute income variables, the coefficient for digital technology decreased the most, indicating that absolute income plays an important role as a mediating variable between digital technology and happiness (Table 8).

### Endogeneity testing of instrumental variables

To address the potential endogeneity issue caused by omitted variables or reverse causality between digital technology and happiness, this paper employs the instrumental variable method. The selection of instrumental variables should adhere to the principles of relevance and exogeneity. This paper selects the digital technology penetration rate at the level of the individual’s community or village, whether there is a computer in the household, and the cost of household communication as instrumental variables, respectively. The estimation results of the two stages of the CMP model are reported in Table 7. From a correlation perspective, the digital technology penetration rate of the location indirectly mirrors the current state of the network infrastructure in the area and impacts an individual’s network usage through “group effect.” The household communication cost indirectly signifies the individual’s digital technology status, while the presence of a computer at home directly decided an individual’s digital technology usage. This paper utilizes the instrumental variable conditional mixed process estimation method (CMP method) to perform the regression analysis. The first-stage regression results of each equatio6 indicate that the impacts of digital technology penetration, household communication costs, and owning a computer at home on digital technology are all significantly positive at the 1% level, satisfying the correlation of instrumental variables. The second-stage regression results reveal that even after adjusting for potential endogeneity bias in digital technology, the impact on happiness remains significant at the 1% level. The effect on happiness is still significantly positive at the 1% level, and the coefficient is significantly larger. This indicates that even after using the CMP method, it is still concluded that digital technology enhance8 happiness.

**Table 6:** Analysis of the influence mechanism.

Variable	(1)	(2)	(3)	(4)	(5)	(6)	(7)	(8)
Digital technology	0.020*** (0.007)	0.022*** (0.007)	0.016** (0.007)	0.026*** (0.007)	0.014* (0.007)	0.011 (0.008)	0.014* (0.007)	0.013* (0.007)
Health status		0.066*** (0.002)						0.047*** (0.002)
Family relations			0.026*** (0.003)					0.009*** (0.003)
Interpersonal relationship				0.087*** (0.001)				0.081*** (0.002)
Non-agricultural work					0.028*** (0.008)			−0.0005 (0.009)
Farming					−0.007 (0.009)			−0.028*** (0.010)
Absolute income						0.025*** (0.003)		0.011*** (0.003)
Relative income							0.063*** (0.003)	0.031*** (0.003)
Control variable	Control	Control	Control	Control	Control	Control	Control	Control
Wald χ2	2364.2***	3324.5***	2429.1***	4454.8***	2375.8***	2290.7***	2550.2***	4636.1***
Observed value	23,465	23,465	23,358	23,446	23,465	21,397	22,662	20,666

The brackets in the table are robust standard errors, and personal characteristics and regional characteristics variables are added to the analysis. ***, **, and * mean significant at 1, 5, and 10% levels, respectively. Control variables in our study conclude personal characteristics and regional characteristics. Among them, “Gender,” “age,” “registered permanent residence,” “nation,” “marital status,” and “education level” were selected as variables to measure personal characteristics. The variables “urban/rural classification” and “province or municipality directly under the central government” were selected to measure regional characteristics. Due to space limitations in the paper, this table list the results for the core variables, do not list the results for the control variables.

### Influence mechanism of digital technology on happiness

In order to substantiate the indirect influence of digital technology on happiness, this study examines its impact on various mediating variables. Table 4 presents the regression coefficients of digital technology in each model, controlling both personal and regional characteristics. In Equation 1, health status is the dependent variable, and the findings indicate that digital technology has a significant negative impact on health status, that is, compared with people who do not use digital technology, digital technology will cause personal health status to deteriorate. Equation 2 takes family relationship as a dependent variable, revealing a significant positive influence of digital technology on family relationships. Through bridging spatial gaps and enhancing connections between individuals and distant relatives or friends via online networks, digital technology fosters harmonious communication among family members, consequently leading to an indirect enhancement of happiness. Equation 3 examines the influence of digital technology on interpersonal relationships, and the outcomes show that digital technology has a significant negative impact on interpersonal relationships. It suggests that digital technology may make interpersonal relationships worse in reality, compared with people who do not use the Internet. Column 4 explores the impact of digital technology on individual employment status, using unemployment as reference group. The results indicate that digital technology actually promotes the probability of individuals engaging in non-farming employment while decreasing the possibility of engaging in farming production. This phenomenon stems from the enhanced job search efficiency and reduced search costs facilitated by online networks. These factors contribute to securing self-employment or wage-based positions, consequently bolstering personal happiness. column 5 examines absolute income as the dependent variable, showing that the regression coefficient of digital technology to absolute income is significantly positive. Specifically, using the network can bring about a significant increase in the *per capita* income o5 families. This outcome is attributed to the ability of digital technology to lower information processing costs, enhance productivity, and facilitate the accumulation of human capital in the realm of information technology. As a result, individuals can command higher wages, thereby elevating their happiness. Column 6 employs relative income as the dependent variable, showing a significant positive association between digital technology usage and relative income. This means that individuals who engage with the internet tend to possess a favorable economic standing within their local community, leading to heightened levels of happiness.

**Table 7:** Estimates of the CMP model.

Variable	(1) Digital Technology Penetration Rate		(2) Household communication costs		(3) Availability of a computer at home	
	Phase I	Phase II	Phase I	Phase II	Phase I	Phase II
Digital technology		0.075*** (0.017)		0.086*** (0.028)		0.080*** (0.024)
Digital technology penetration	0.595*** (0.015)					
Household communication costs			0.044*** (0.003)			
Availability of a computer At home					0.114*** (0.005)	
Control variable	Control	Control	Control	Control	Control	Control
Wald χ^2^	5820.34 ***	4107.02 ***	6018.88 ***	4087.22 ***	5940.80 ***	4020.90 ***
Observed value	20,664	20,664	20,664	20,664	20,664	20,664

Robust standard errors are in parentheses in the table. Mediating and control variables are added to the analysis. ***, **, and * indicate significant at the 1, 5, and 10% levels, respectively. Control variables in our study conclude personal characteristics and regional characteristics. Among them, “Gender,” “age,” “registered permanent residence,” “nation,” “marital status,” and “education level” were selected as variables to measure personal characteristics. The variables “urban/rural classification” and “province or municipality directly under the central government” were selected to measure regional characteristics. Due to space limitations in the paper, this table list the results for the core variables, do not list the results for the control variables.

There may be several influence channels for digital technology to influence happiness. To further elucidate the mechanisms, this study incorporates intermediary variables into the benchmark model to investigate the pathways through which digital technology influences happiness. The results of the regression analysis are presented in Table 6. Equation 1 represents the benchmark model. After adjusting for variables such as individual characteristics and regional traits, the coefficient for digital technology is 0.020. Equation 2 adds health status variables to the benchmark model, and reveals a significant positive association between health status and happiness. Subsequently, the marginal effect of digital technology increases by 10%, with the coefficient rising to 0.022 and achieving statistical significance at the 1% level. This suggests that, upon controlling health status variables, the impact of digital technology on happiness intensifies, hinting at a possible negative relationship between personal health status and digital technology, indicating that engagement with digital technology may affect individual health outcomes. In the baseline model, the inclusion of family relationship variables in the third column of equations results in a significant positive impact of family relationships on happiness, while the marginal effect of digital technology decreases to 0.016 but remains significant. This suggests a positive correlation between family relationships and digital technology, as communication means such as networks increase the frequency of interaction with non-cohabiting family members, thereby promoting harmony in family relationships and then increasing happiness. Adding interpersonal variables to the fourth equation shows that the interpersonal relationship coefficient is significantly positive, that is, the improved interpersonal connections can boost happiness. However, the coefficient for digital technology also rises, indicating a negative association between interpersonal relationships and digital technology. This implies that digital technology may hamper real-life interpersonal communication, subsequently impacting happiness adversely. The variable of employment status is introduced into the fifth equation.

**Table 8:** Robustness tests of 2018 CFPS data.

Variable	(1)	(2)	(3)	(4)	(5)	(6)	(7)	(8)
Digital technology	0.032*** (0.007)	0.035*** (0.007)	0.027*** (0.007)	0.023*** (0.007)	0.027*** (0.008)	0.017** (0.008)	0.021*** (0.008)	0.011* (0.006)
Health status		0.072*** (0.002)						0.049*** (0.003)
Family relations			0.019*** (0.002)					0.009*** (0.002)
Interpersonal relationship				0.155*** (0.003)				0.142*** (0.003)
Non-farming job					0.036*** (0.006)			−0.008 (0.006)
Farming job					0.011*(0.006)			−0.003 (0.006)
Absolute income						0.053*** (0.003)		0.027*** (0.003)
Relative income							0.066*** (0.002)	0.037*** (0.003)
Control variable	Control	Control	Control	Control	Control	Control	Control	Control
Wald χ^2^	1934.4***	2663.5***	2047.5***	4496.8***	1971.1***	2135.4***	2480.5***	4821.5***
Observed value	26,654	26,654	26,569	26,621	26,654	24,819	25,272	23,556

Robust standard errors are in parentheses in the table, ***, **, and * indicate significant at the 1, 5, and 10% levels, respectively. Control variables in our study conclude personal characteristics and regional characteristics. Among them, “Gender,” “age,” “registered permanent residence,” “nation,” “marital status,” and “education level” were selected as variables to measure personal characteristics. The variables “urban/rural classification” and “province or municipality directly under the central government” were selected to measure regional characteristics. Due to space limitations in the paper, this table list the results for the core variables, do not list the results for the control variables.

The results show that if the unemployed are taken as the reference group, the probability of happiness of individuals with non-agricultural jobs is obviously higher, while farming has no significant effect on happiness. Increasingly, the marginal effect of digital technology decreases by 30% in this scenario, with the coefficient dropping to 0.014 and maintaining significance at the 10% level. This means that digital technology influences individual employment statuses, subsequently impacting happiness levels. Adding the variable of absolute income to the column 6, the coefficient of absolute income is significantly positive, while the coefficient of digital technology decreases to 0.011 and is not significant. This indicates that absolute income mediates the relationship between digital technology and happiness, as digital technology positively affects happiness by increasing individuals’ absolute income. When the variable of relative income is introduced into the column 7, the coefficient of relative income is also significantly positive, that is, individuals with higher relative income within their local area tend to report higher happiness levels. Meanwhile, the marginal effect of digital technology diminishes, implying a positive correlation between digital technology and relative income. This suggests that individuals with access to digital technology often possess relatively higher incomes within their local regions, contributing to their heightened happiness. After controlling the intermediate variables in the eighth equation, the marginal effect coefficient of digital technology becomes 0.013, retaining significance at the 10% level. This signifies that digital technology can directly enhance happiness, apart from its influence through various intermediary channels. Based on the above analysis, the inclusion of interpersonal relationships, employment status, absolute income, and relative income in the benchmark model leads to a significant alteration in the digital technology coefficient of over 30%. This underscores the notion that digital technology can indirectly impact happiness by influencing interpersonal communication, employment, income dynamics, and especially by affecting individuals’ absolute income, thereby exerting a substantial influence on overall happiness levels.

## Conclusion and discussion

This article reviews the relevant literature on digital technology and happiness. Based on the 2020 China Family Panel Studies (CFPS) data, an ordered probit model is constructed to test the impact of digital technology on happiness, and explores the robustness of the happiness effect and use CMP model to test the endogeneity. Then, the analysis includes an exploration of the heterogeneity of “happiness effect” across various demographic characteristics such as gender, age, education and registered population status. Finally, the paper selects health status, interpersonal relationship, employment status and income level as intermediary variables to explore the influence mechanism of digital technology on happiness.

Based on empirical analysis, the results show that: Firstly, digital technology can improve personal happiness. The marginal effect coefficient of digital technology is 0.031. Secondly, this paper use the CMP model of instrumental variables to confirm the substantial positive relationship between digital technology and happiness. Thirdly, digital technology has a heterogeneous effect on personal happiness, especially for women, young people, primary school and college graduates and rural residents. With the increase of absolute income, the happiness effect of digital technology is decreasing. It can also be said that digital technology reduces the promotion of absolute income to happiness. Fourthly, in terms of influencing mechanism, digital technology can indirectly affect individual happiness by influencing health status, interpersonal relationship, employment situation and income level. Specifically, digital technology is found to have a negative effect on personal health, interpersonal relationships and agricultural work, while positively affecting family relationships, non-farming employment, absolute income, and relative income. This leads to an increase in the likelihood of individuals engaging in non-farming employment and a decrease in the likelihood of farming activities. Overall, the study suggests that digital technology can indirectly influence happiness by affecting these intermediary variables. Empirical tests further reveal that digital technology has the most pronounced effect on happiness through its impact on increasing absolute income.

Digital technology not only directly impacts happiness by enhancing entertainment options, broadening information access, and facilitating convenience in daily life, but also has an indirect effect on happiness by influencing personal health, family dynamics, interpersonal communication, work status, and income levels. Although the penetration rate of digital technology increasing year by year, there are still problems with weak network infrastructure in rural areas and weak network skills among rural residents. According to the report of *China Internet Network Information Center* (CNNIC), the Internet penetration rate in rural areas of China in 2020 is 55.9%, which is significantly lower than 79.8% in urban areas, and 51.5 and 13.3% residents, respectively, indicate that the reason for not going online is because they do not understand the network and do not have Internet access equipment. It is of great significance to increase network technology training and strengthen network infrastructure construction to increase the penetration rate of digital technology. Based on the above analysis, this paper proposes that: On the one hand, it is crucial to strengthen the capacitation of digital skills, particularly among vulnerable demographics such as the elderly and rural inhabitants, in order to elevate their proficiency in digital technologies. This can be achieved through the integration of additional educational resources, thereby facilitating the provision of cost-free digital technology training and guidance. By doing so, a broader spectrum of individuals will be able to partake in the happiness dividends entailed by the era of the network economy. On the other hand, it is worth noting that the improvement in digital infrastructure is often related to regional economic and social factors, such as government projects specifically for the purpose of increasing access to digital technology or related digital industry policies. The digital infrastructure discussed in this paper focusing on the equipment that can directly affect the Internet access of rural residents in remote areas, such as high-speed Internet broadband, network signal towers, PC computers or mobile communication equipment, etc. The investment of these facilities can increase the use rate of digital technology for rural residents in remote areas. According to the analysis above, government should improve the construction of digital infrastructure and provide digital technology access equipment for rural residents in remote areas, such as high-speed Internet broadband, network signal towers, PC computers or mobile communication equipment, these facilities can directly enhance their digital technology usage. At the same time, it is necessary to combine industrial policies, give full play to the employment effect of digital infrastructure, establish Internet manufacturing factories in the urban–rural fringe or distant suburbs, and provide more high-quality jobs with high incomes for rural workers to improve their happiness.

In summary, the low utilization rate of digital technology is the result of multiple factors such as lack of digital infrastructure and digital skills training, solving these problems requires joint efforts from the government and all sectors of society, gradually narrowing the digital divide between urban and rural areas through various measures such as improving infrastructure, enhancing education levels, and promoting digital skills training. It is worth noting that in reality, individual happiness is influenced by health status and interpersonal relationships, and is also closely related to personal employment, income level and job satisfaction. Therefore, emphasis should be placed on promoting network education and values, cultivating healthy online habits, mitigating excessive internet usage which may adversely impact physical and mental well-being, and fostering positive life and interpersonal communication practices. Such efforts will enable digital technology to serve as a vital tool for enhancing personal welfare and fostering happiness in the digital economy era.

## Data Availability

The original contributions presented in the study are included in the article/supplementary material, further inquiries can be directed to the corresponding author/s.
